# Supplementary material for: In vivo genome editing via CRISPR/Cas9-mediated homology-independent targeted integration for Bietti crystalline corneoretinal dystrophy treatment
Source: Nat Commun. 2024 May 6;15:3773. doi: 10.1038/s41467-024-48092-9 (PMC11074121; doi:10.1038/s41467-024-48092-9)
Supplement: Supplementary file 1 — Supplementary Information [file 41467_2024_48092_MOESM1_ESM.pdf]

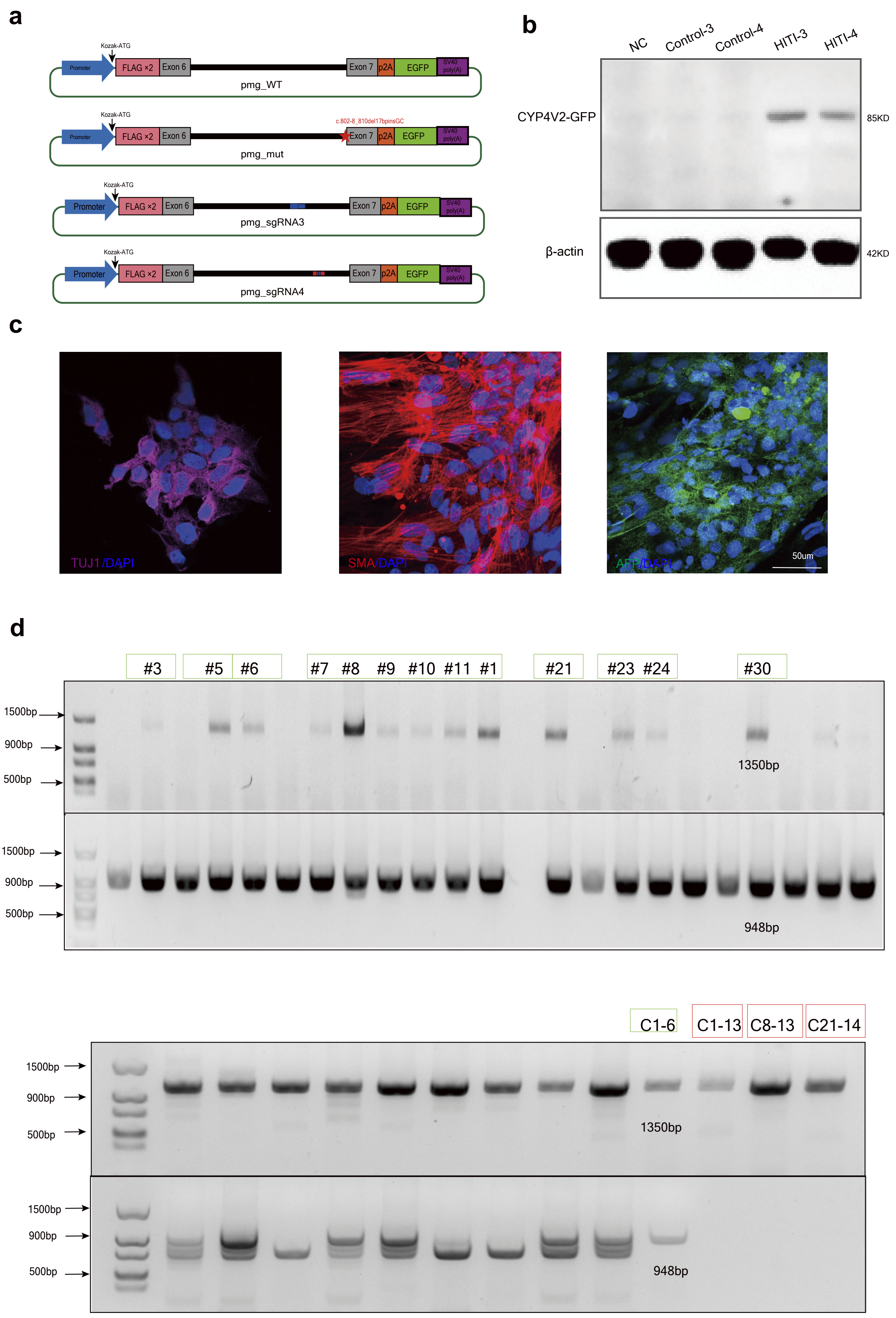
a
Kozak-ATG
Promoter
FLAG ×2
Exon 6
Exon 7
p2A
EGFP
SV40 poly(A)
pmg_WT
c.802-8_810del17bpinsGC
pmg_mut
pmg_sgRNA3
pmg_sgRNA4
b
NC
Control-3
Control-4
HITI-3
HITI-4
CYP4V2-GFP
85KD
β-actin
42KD
c
TUJ1/DAPI
SMA/DAPI
AFP/DAPI
50um
d
#3
#5
#6
#7
#8
#9
#10
#11
#1
#21
#23
#24
#30
1500bp
900bp
500bp
1350bp
948bp
C1-6
C1-13
C8-13
C21-14

Supplementary Figure 1. *In vitro* genome editing using the HITI-based method.

a Schematic diagram of minigene plasmid. Blue rectangle indicates stub crRNA sequences; Red rectangle indicates PAM sequence. **b** Western blot results showed that the repaired sequence can be effectively transcribed and translated. CYP4V2-GFP were detected with anti-GFP antibody, indicating repaired CYP4V2. **c** Immunofluorescence staining of three germ-layer markers TUJ1, SMA and AFP in embryoid body (EB)-based differentiation of iPSCs colony. Scale bar=50μm. **d** Agarose gel picture in process of screening edited iPSCs. 1350 bp indicates PCR product from edited genomes, 948bp indicates PCR product from unedited genomes. Green rectangle indicates heterozygous clones and red rectangle indicates homozygous clones. Source data are provided as a Source Data file.

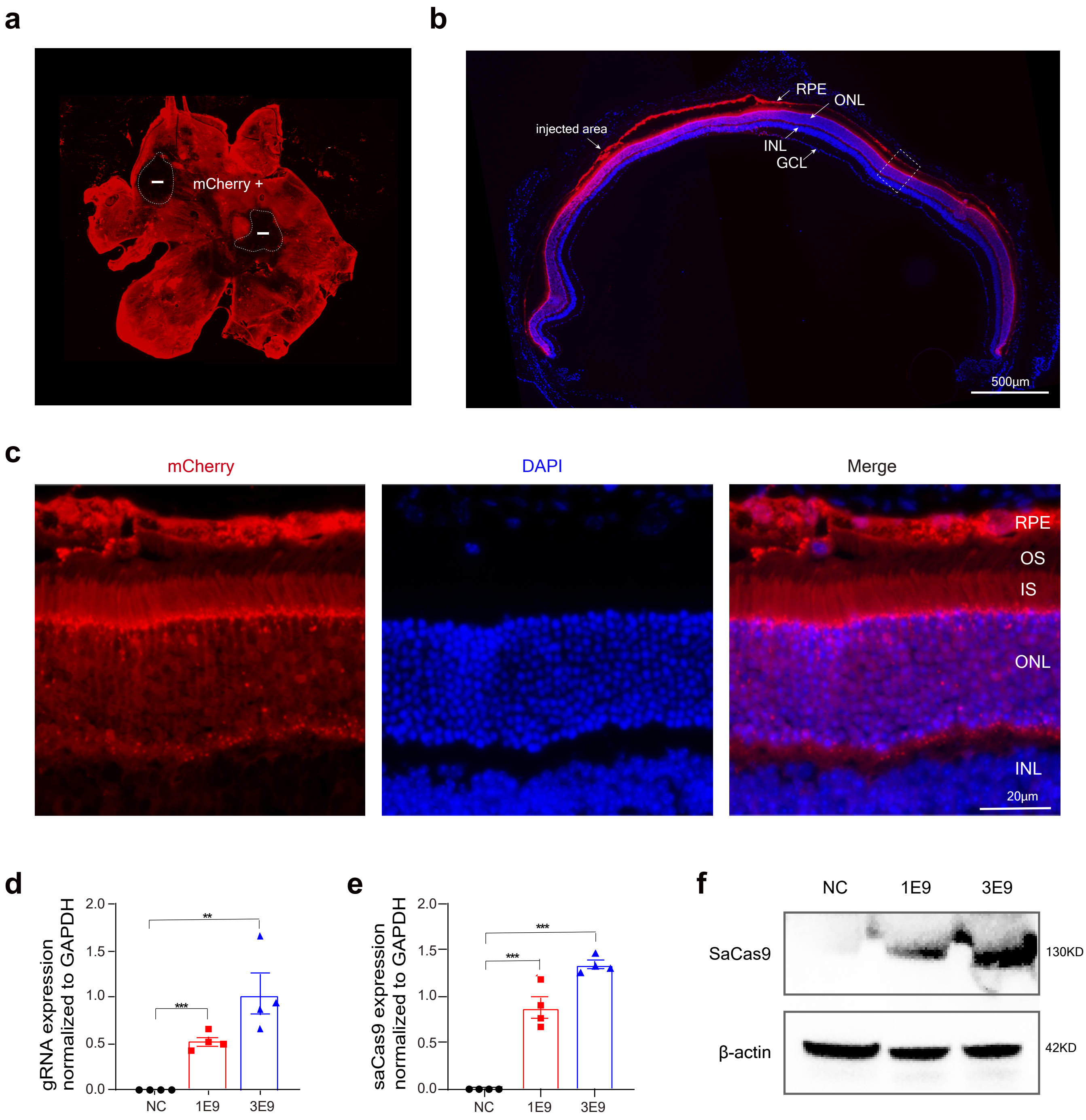

Supplementary Figure 2. AAV vectors transduction profile and Cas9 mRNA and gRNA expression in the retina.

a Whole mount RPE-choroid complex of mouse post-injection 1 month at 3×10^{9} dose showed the scope of AAV8 transduction. The mCherry expressed negative (-) in the flat of lacking RPE cells due to stretching during preparation (top left) and the mount without transduced (low right). **b-c** Full-view retinal sections (b) and zooming in for higher power images of rectangle zone (c) showed the transduced retinal areas. RPE, retinal pigment epithelium; OS, outer segments; IS, inner segments;

ONL, outer nuclear layer; INL, inner nuclear layer. GCL, ganglion cell layer. Scale bars: 500μm(b); Scale bars: 20μm(c). **c-d** Quantitative PCR analysis indicated the expression of gRNA (d) and SaCas9 (e) in two doses groups at 5 months after treatment (*n*=4 eyes/group). Student's unpaired two-tailed t-test. Error bars represent the mean ± SEM. ***p*<0.01, ****p*<0.001. **f** Determination of SaCas9 expression by Western blot in two doses groups at 5 months after treatment. NC, negative control, represent *h-Cyp4v3*$^{mut\ mut}$ mice without treatment; 1E9 represent *h-Cyp4v3*$^{mut/mut}$-1E9 mice, 3E9 represent *h-Cyp4v3*$^{mut/mut}$-3E9 mice. Exact P values: (d) NC vs. 1E9 *p*=2.5e-5, NC vs. 3E9 *p*= 0.00351. (e) NC vs. 1E9 *p*=0.000260, NC vs. 3E9 *p*= 1.348e-7. Source data are provided as a Source Data file.

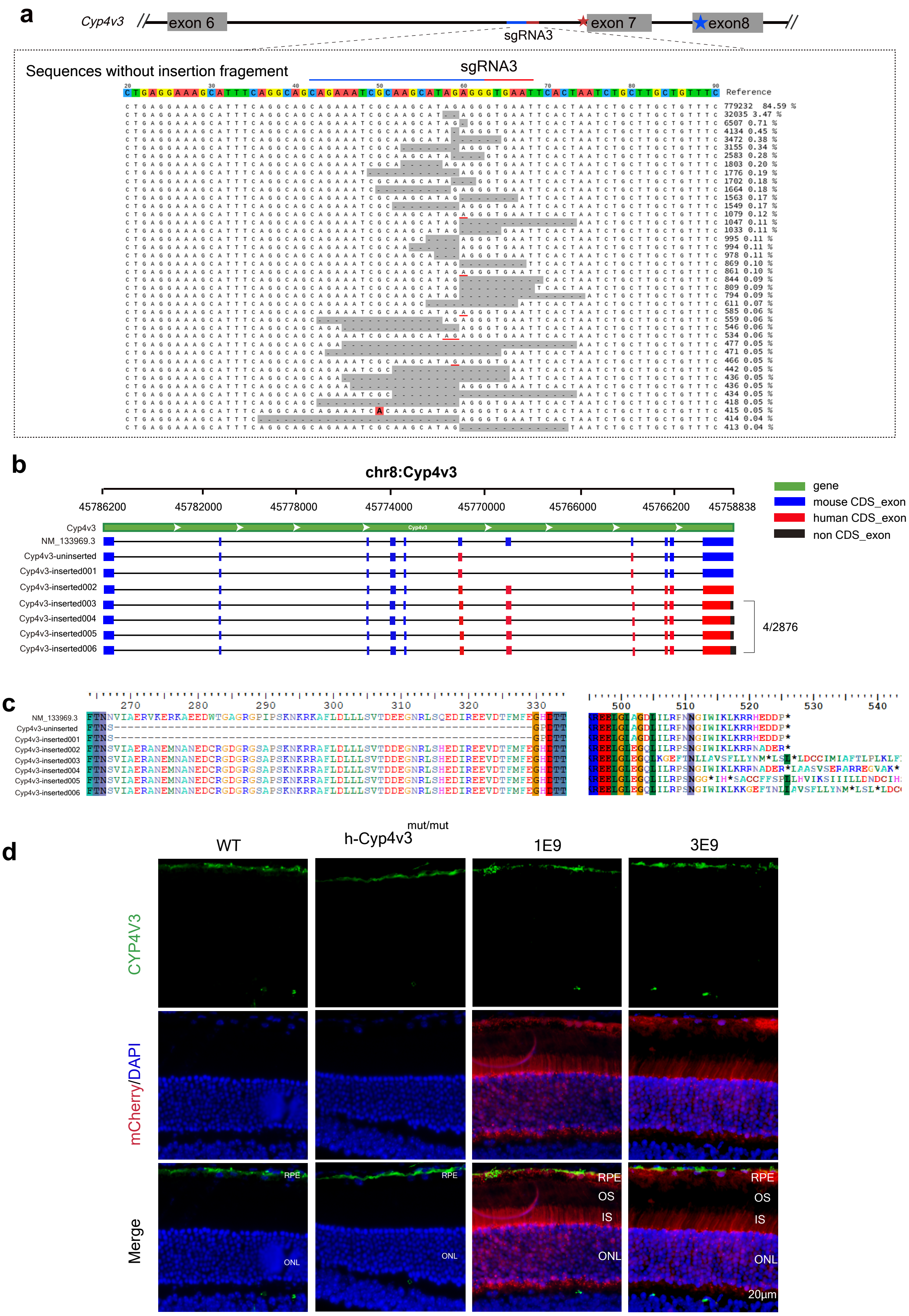
a
Cyp4v3
exon 6
sgRNA3
exon 7
exon8
Sequences without insertion fragement
sgRNA3
Reference
b
chr8:Cyp4v3
45786200
45782000
45778000
45774000
45770000
45766000
45766200
45758838
gene
mouse CDS_exon
human CDS_exon
non CDS_exon
Cyp4v3
NM_133969.3
Cyp4v3-uninserted
Cyp4v3-inserted001
Cyp4v3-inserted002
Cyp4v3-inserted003
Cyp4v3-inserted004
Cyp4v3-inserted005
Cyp4v3-inserted006
4/2876
c
NM_133969.3
Cyp4v3-uninserted
Cyp4v3-inserted001
Cyp4v3-inserted002
Cyp4v3-inserted003
Cyp4v3-inserted004
Cyp4v3-inserted005
Cyp4v3-inserted006
d
WT
h-Cyp4v3 mut/mut
1E9
3E9
CYP4V3
mCherry/DAPI
Merge
RPE
OS
IS
ONL
20μm

Supplementary Figure 3. HITI-based method achieves precise genome editing, transcription and translation in *h-Cyp4v3*$^{mut/mut}$.

a PCR-based NGS analysis for those sequences without *CYP4V2* donor insertion in *h-Cyp4v3*$^{\mathrm{mut/mut}}$ mice after treatment. **b-c** Prediction of transcripts (b) and translations (c) of total sequences, * indicates the stop codon. **d** IF staining indicates CYP4V3 expression in mouse retina of wild type, *h-Cyp4v3*$^{mut/mut}$, *h-Cyp4v3*$^{mut/mut}$-1E9/3E9 mice. RPE, retinal pigment epithelium; OS, outer segments; IS, inner segments; ONL, outer nuclear layer; INL, inner nuclear layer. Scale bars: 20μm. Source data are provided as a Source Data file.

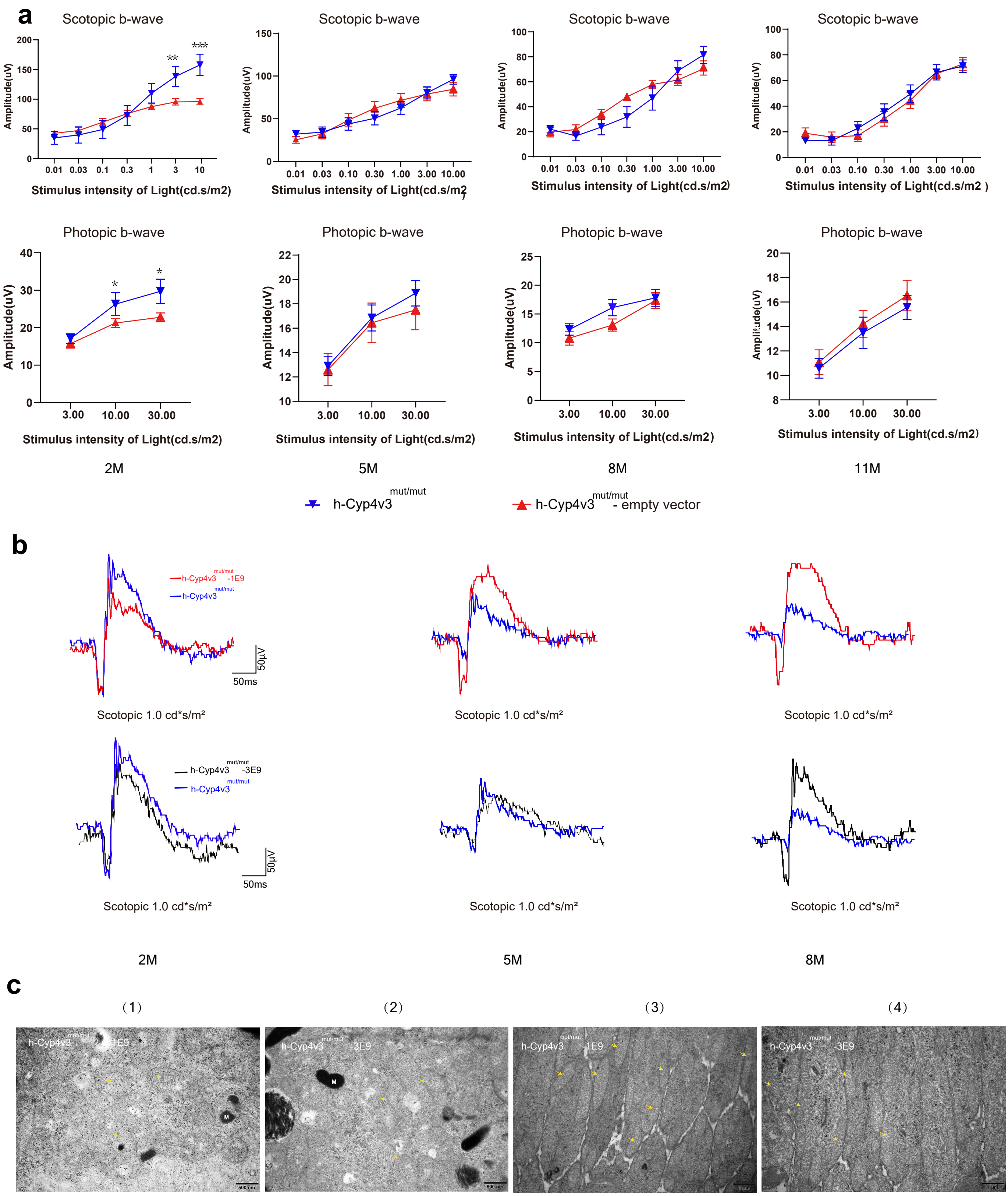

Supplementary Figure 4. HITI-based method restores visual function and retinal degeneration.

a Statistic graph of ERG amplitude of scotopic/photopic b-wave in *h-Cyp4v3*$^{mut\ mut}$-empty vector mice at 2, 5, 8, 11 months after treatment (*n*=27, 19, 8, 10 eyes) and age-matched *h-Cyp4v3*$^{mut/mut}$ mice (*n*=17,

34, 14, 11 eyes). Student's unpaired two-tailed t-test. Error bars represent the mean ± SEM. Exact P values: (3.0 cd*s/^{m2} and 10.0 cd*s/^{m2} scotopic b-wave at 2 months after treatment) p=0.00617, p=0.000311, (10.0 cd*s/^{m2} and 30.0 cd*s/^{m2} photopic b-wave at 2 months after treatment) p=0.0498 p=0.0238. *p<0.05, **p<0.01. **b** Representative scotopic (intensity of 1.0 cd*s/m^2) ERG waveforms of *h-Cyp4v3$^{mut/mut}$*-1E9 and *h-Cyp4v3$^{mut/mut}$*-3E9 mice at 2,5 and 7 months after treatment. **c** TEM images of *h-Cyp4v3$^{mut/mut}$*-1E9/3E9 mice at 11 months after treatment, the results showed that mitochondria appeared normal in IZ (**e1-2**) and EZ (**e3-4**) band of photoreceptor with increased lamellar cristae, indicated with yellow arrowheads (**e1-2**), scale bars: 500nm(**e3-4**), scale bars: 1µm. Source data are provided as a Source Data file.

Supplementary Table 1. Primers used in this study

Catalogues		Sequences
Primers designed for cDNA of 293T cells after transfected with minigene plasmids	F	cctggctttggcttgatctct
	R	cagggttctcctccacgtct
Flanked on edited genomic region in iPSCs	F	fggaggtctctctgatcgtgt
	R	caactagaaggcacagtcagc
Flanked on unedited genomic region in iPSCs	F	cagtgctagggacaggggta
	R	gtgcccagcactagtcaaaatc
Flanked on edited cDNA of *CYP4V2*	F	gtttttagaaccatggcttggcct
	R	taacctgccacttcacaatcttca
Primers to confirm the genome of the h-*Cyp4v3*$^{mut/mut}$ mouse	F1	aagagcaatcggcaacattt
	F2	tgaacaaacagggtgctgtg
	F3	aggccacttagtagcaggtt
	F4	agtttcaccagataatcccc
	F5	atgcaagtccacttcctcac
	F6	ctaacatgctgggaccatga
	F7	gcacacataaatgcacacag
	F8	ctgcaagtgctaggaaaatg
	F9	gtccttactgcacagggtgt
Primers to confirm the cDNA of *Cyp4v3* in the h-*Cyp4v3*$^{mut/mut}$ mouse	F	atgttgtggctgtggttaggg
	R	gcctcaggatacaggcaaga
Primers for *Pde6b*^{rd1}mutation detection	F	tacccacccttcctaatttttctcagc
	R	tgacaattactccttttccctcagtctg
Primers for *Crb1*^{rd8} mutation detection	F	gtgaagacagctacagttctgatc
	R	gccccatttgcacactgatgac
Primers for *Pde6b*^{rd10} mutation detection	F	acaaggaacaagggctctga
	R	ccttccactcattgctaggac
Primers for *Rpe65*^{rd12} mutation detection	F	tgacacctagttttaatattttgatcc
	R	cagagctcttgaaccccatt
Primers for quantitative PCR of Cas9 mRNA expression	F	ccgcccggaaagagattatt
	R	cggagttcagattggtcagtt
SaCas9 Probe		agctgctggatcagattgccaaga
Primers for quantitative PCR of GAPDH expression	F	ccaaggtcatccatgacaact
	R	ggccatccacagtcttctg
GAPDH Probe		atgaccacagtccatgccatcact
Primers for quantitative PCR of gRNA mRNA expression	F	cagaaatcgcaagcatagagg
	R	ttgacgagataaacacggcattt
sgRNA probe		agataaacacggcattttgccttgtt
Flanked on mE9, mE10(unedited sequence) in h-*Cyp4v3*$^{mut/mut}$ mouse after treatment	F	cttagcgaggactgtgaagtgg
	R	gaaagaaccgctctggtcggaa
Flanked on hE10, hE11(edited sequence) in h-*Cyp4v3*$^{mut/mut}$ mouse after treatment	F	agttccagcctgagcggttctt
	R	cctcaggatgcacgaaagaatgg
Flanked on mE2, hE9 of edited fusion cDNA in h-*Cyp4v3*$^{mut/mut}$ mouse after treatment	F	tcgacacctgccgatcatta
	R	aaggaaaaaggcgaagggtct
T7E1 assay primers of sgRNA1,5	F	gtgaagtacaccctaccccca
	R	ccaggtaattgaccaaacctgc
	F	ggatgggaacacaaaaagagcc

T7E1 assay primers of sgRNA2,6,7	F	ggatgggaacacaaaaagagcc
	R	acacagcaccctgtttgttcata
T7E1 assay primers of sgRNA3,4	F	aagcatggcagtgtttgagttg
	R	cgttcatttcattggcccgt
Sequencing at 5'junction of insertion in HEK293T cells	F	agggaggtctctctgatcgtg
	R	ctccgcttccgcgttcatct
Sequencing at 3'junction of insertion in HEK293T cells	F	tgcccgacaaccactacct
	R	acttagcctgttcccttcgtc
Sequencing at 5'junction of insertion in posttreatment h-*Cyp4v3*$^{mut/mut}$ mouse	F	ggaggtctctctgatcgtgt
	R	caactagaaggcacagtcagc
Sequencing at 3'junction of insertion in posttreatment h-*Cyp4v3*$^{mut/mut}$ mouse	F	tctaaaaggcactgaagccgt
	R	tgcttgggaaaactctgacaa
Sequences without successful insertion in posttreatment h-*Cyp4v3*$^{mut/mut}$ mouse	F1	ggctctaggaattccaccaaga
	R1	aactcggatcgaaagaccca
	F2	ggggcagccgtgcaggcctct
	R2	tagaggagaaaagaaacagca

Supplementary Table 2. List of In Silico Predicted Candidate Off-Target Sites and NGS Analysis of sgRNA3

	Category	PAM coordinate	Strand	Deletions	Insertions	InDels	Modified	Unmodified
On target		chr4:186200708		123826(25.1813%)	9409(1.9134%)	4912(0.9989%)	138147(28.0936%)	353591(71.9064%)
Off target 1	3 mismatches + bulge (RNA)	chr1: 158843376	+	0(0.0000%)	641(0.3569%)	4(0.0022%)	645(0.3591%)	178972(99.6409%)
Off target 2	3 mismatches + bulge (DNA)	chr1: 208281165	-	1(0.0004%)	617(0.2685%)	3(0.0013%)	621(0.2702%)	229213(99.7298%)
Off target 3	3 mismatches + bulge (DNA)	chr2: 14039378	+	1(0.0006%)	542(0.3113%)	17(0.0098%)	560(0.3216%)	173575(99.6784%)
Off target 4	3 mismatches + bulge (RNA)	chr2: 195141060	+	2(0.0010%)	1371(0.7075%)	14(0.0072%)	1387(0.7158%)	192394(99.2842%)
Off target 5	3 mismatches + bulge (RNA)	chr2:223912855	+	0(0.0000%)	310(0.2867%)	26(0.0240%)	336(0.3107%)	107808(99.6893%)
Off target 6	3 mismatches + bulge (RNA)	chr2:232114706	+	0(0.0000%)	267(0.2799%)	16(0.0168%)	283(0.2966%)	95119(99.7034%)
Off target 7	3 mismatches + bulge (RNA)	chr3 :3188480	-	1(0.0009%)	294(0.2556%)	4(0.0035%)	299(0.2600%)	114720(99.7400%)
Off target 8	3 mismatches + bulge (RNA)	chr3 :85567070	-	0(0.0000%)	1214(0.6227%)	0(0.0000%)	1214(0.6227%)	193759(99.3773%)
Off target 9	3 mismatches + bulge (RNA)	chr4: 45040319	-	2(0.0028%)	155(0.2153%)	36(0.0500%)	193(0.2681%)	71787(99.7319%)
Off target 10	3 mismatches + bulge (RNA)	chr4: 94637893	+	5(0.0030%)	3193(1.8880%)	13(0.0077%)	3211(1.8986%)	165911(98.1014%)
Off target 11	3 mismatches + bulge (RNA)	chr5 :66558069	-	0(0.0000%)	2483(1.1928%)	3(0.0014%)	2486(1.1942%)	205684(98.8058%)
Off target 12	3 mismatches + bulge (RNA)	chr5 :113084094	-	0(0.0000%)	520(0.3869%)	9(0.0067%)	529(0.3936%)	133866(99.6064%)
Off target 13	3 mismatches + bulge (RNA)	chr5: 133390863	+	0(0.0000%)	1391(0.6822%)	14(0.0069%)	1405(0.6891%)	202498(99.3109%)
Off target 14	3 mismatches + bulge (RNA)	chr5: 159737753	-	2(0.0010%)	672(0.3214%)	63(0.0301%)	737(0.3525%)	208327(99.6475%)
Off target 15	3 mismatches + bulge (RNA)	chr6: 6832307	+	0(0.0000%)	769(0.2808%)	0(0.0000%)	769(0.2808%)	273047(99.7192%)
Off target 16	3 mismatches + bulge (RNA)	chr8:35858985	-	2(0.0007%)	810(0.2730%)	19(0.0064%)	831(0.2801%)	295865(99.7199%)
Off target 17	3 mismatches + bulge (RNA)	chr8: 106213049	-	4(0.0027%)	461(0.3058%)	46(0.0305%)	511(0.3389%)	150262(99.6611%)
Off target 18	3 mismatches + bulge (RNA)	chr8: 134469950	+	1(0.0004%)	570(0.2420%)	19(0.0081%)	590(0.2505%)	234923(99.7495%)
Off target 19	3 mismatches + bulge (RNA)	chr10: 105383691	+	3(0.0033%)	143(0.1553%)	57(0.0619%)	203(0.2204%)	91891(99.7796%)
Off target 20	3 mismatches + bulge (DNA)	chr10: 117989847	+	5(0.0025%)	716(0.3573%)	2(0.0010%)	723(0.3608%)	199649(99.6392%)
Off target 21	3 mismatches + bulge (RNA)	chr14: 56101256	-	0(0.0000%)	664(0.2716%)	0(0.0000%)	664(0.2716%)	243781(99.7284%)
Off target 22	3 mismatches + bulge (RNA)	chr14: 86889989	+	0(0.0000%)	433(0.2025%)	31(0.0145%)	464(0.2170%)	213392(99.7830%)
Off target 23	3 mismatches + bulge (RNA)	chr18: 58732216	+	4(0.0028%)	437(0.3030%)	18(0.0125%)	459(0.3183%)	143762(99.6817%)
Off target 24	3 mismatches + bulge (RNA)	chr18: 60189498	+	0(0.0000%)	519(0.2258%)	7(0.0030%)	526(0.2288%)	229357(99.7712%)

Supplementary Table 3. List of Predicted Off-Target Sites and Primers for Sanger Sequencing Analysis

	PAM coordinate	Strand	Mis-matches	Bulge Size	Sequence	Primer-F	Primer-R
On target	chr8：45317698						
Off target 1	chr1：43043561	+	3	1	AGTcAGGACGGGAaCtCAAAAAGG	GCTTTGAAACCCACTCCCCT	CCGGCAACCACACTCTTCTT
Off target 2	chr1：39420431	+	3	1	AGACAGGAtGGGACCtCAAAcAGG	ACCACCCCCTTAACCCTTTC	CCAATGCTCATGACAACACGG
Off target 3	chr2：120332980	+	3	1	AGGAAGGAgaGGACaACAAAAGGG	GGTAGGGGCAACAGCTAAGG	GGGTGGATGCTACCCTTGAAT
Off target 4	chr3：112059902	-	3	0	AGAAaGAaGGGACaACAAAAGGG	AGTTTCAGGAGATCCAGTGCC	CTGTGAGGACAGCAAGATGGT
Off target 5	chr3：117424505	-	3	0	AGAAGaAgGGGAtCACAAAAGGG	TGCTAATTTTGGAATTTGGGGC	CTGGCCACCCAGTACTACCA
Off target 6	chr5：126181863	-	3	0	AGAAGaACaGGACCACAAAgAGG	GGGGCCCACTGGCTAATTTT	CTGGGGCAGAGGGATAAATGA
Off target 7	chr5：32212183	+	3	1	AGACAGGACaGGAgCtCAAAACGG	AGCTTTGACCGAATGAGCACT	GGAACAGAGCTGAGCGTGAC
Off target 8	chr6：128055315	-	3	0	AGAAGcAaGGGACCcCAAAAGGG	CCCATTAGAATGCCCTCGCA	GACCAGTCTCCACTGATGCC
Off target 9	chr6：7250660	+	3	1	AGGAAGGAatGGACCACtAAAGGG	TTGTATGGACTCCCAGACCCA	CTTTTCCCCCATAGCTGCAC
Off target 10	chr8：88672248	-	3	1	AGcAAGGACaaGACCACAAAAAGG	GTAGCACAGGCTGTTGGACT	TGCTCAGCTGCCCCTAAATC
Off target 11	chr8：104292984	-	3	1	AGAGAGGACacGACCcCAAAAAGG	TTCCCTATTTCCGAGGACAGC	GGTGGGGCTCAGTGAATGCT
Off target 12	chr9：122555633	-	3	0	AGAAaGACaGGACCAgAAAATGG	TCTGCAAGCACCCTTGTTCA	TGAGCAACGCCTTAGTCTGG
Off target 13	chr10：77931278	-	3	0	AGAAaGAgGGGACCcCAAAAAGG	CCACAGTTAGGGAGGCCAAC	AAAGACTTGGGCCGATGGAG
Off target 14	chr11：32790257	-	3	0	AGAAGtttGGGACCACAAAATGG	GGCTCTTCTCTGCCCTCATT	CACCCTCAGGTTCAACTCTCC
Off target 15	chr14：34977570	-	3	1	AGAAAGGACaGGAaCcCAAAAGGG	ATGGGCGTCGATACTCTCCA	GCACGGTTAAGTGTGGTCAG
Off target 16	chr15：13578201	-	3	1	AGGAAaGACGGcACaACAAAAAGG	GCAGACAAATGAAGCTTGGGG	GCCAAGTCACGAGGTACTGAA
Off target 17	chr15：73882315	-	3	1	AGcAAGtACaGGACCACAAAAGGG	TCAGTGTATGCTGTGCACCC	CTCCCTACATCAGCACCCAG
Off target 18	chr16：46544138	-	3	0	AGAAGGAaGGaACtACAAAAAGG	GGTGAACTGACACCCGACTT	ATCAGGTTTCCCACAGGCAG
Off target 19	chr16：12022267	+	3	1	AGGAAGGACaGGACCACAtcAAGG	GCCCTCTACCACTTACCACG	TGTAAAGGCAGCACCTGTCC
Off target 20	chr17：46927335	+	3	1	AGGgAGGAgGGGACCACAAAgGGG	TCCCGATTTGTGGACATGGG	CTTCTACACCCACCCGACAC
On target	chr8：45310221						
Off target 21	chr1：77616545	+	3	1	CTTCTGGgTTCGTttCCAACTTGG	ATGCACAGCATAGACACCACAAT	ATGGCTTTCTTCAGTGAGGCT
Off target 22	chr2：120010090	+	3	1	CTATCTGGATTCGatCCCAAcTGG	GTCTGCTGTTCCACCGGTAA	AGAGTGTATATGCGCGTCCC
Off target 23	chr3：75864394	+	3	1	CTTCAgtGATTCGTGCCCAgTAGG	GTGGGCTGCTCCTTTCTTTTG	TTGTGGCCATACAACCAGTG
Off target 24	chr4：151623884	-	3	1	CTTCTAGGAcTtGTGCCCAAgTGG	TTCCACAGGAAGAACCGAGG	AGATGACTGGCAAGTGGGTC
Off target 25	chr6：57763692	-	3	1	aTGTCTGGATTCGTGtCaAATTGG	AGGCCACTTCCAGGCAATAC	TGCCATTCAGTTTTCACCGC
Off target 26	chr7：87450024	-	3	1	CTTTCTGaATaCtTGCCCAATCGG	GGCAGCCAAAACAGACTCAAG	GTGAGGACCTTAGTGAGAGGG
Off target 27	chr8：44895971	+	3	0	CTTCTGGATTCGTGtCCtAgAGG	TCAGGCACCGTTCTTGGATT	AGGGACAAGACAGTGTGGGA
Off target 28	chr8：49444529	-	3	1	CTTCTGGAAcTCGgGCCCAAgAGG	GAGGAACGACTTCCGTCAGG	AAAGGTGACGCATTGGCAAC
Off target 29	chr9：16225054	-	3	1	CTTCTGGACTTCtgaCCCAATGGG	GATGGGAGGGGAAAGCCCTA	TCCCATGTCCCACTAATGTGC
Off target 30	chr10：23418076	+	3	0	CTTCTGaATTtGTtCCCAATAGG	TTTGTGTGGGCTGCTGTACT	AGGGGCTACTCTAGGCTCTG
Off target 31	chr10：117515610	-	3	0	CTTCTGtATTCcTGCaCAATGGG	AGAGGCATTTGGTCCCATCC	ACTGCATGACACCCGTCTTT
Off target 32	chr11：117341369	-	3	1	CTTCTGGAAgctGTGCCCAATAGG	CTAAGCCCGGAGAGACAAGC	CAGTAACTGCACTGCCCCAT
Off target 33	chr11：6670115	+	3	1	CTTCTGGATTCAtTGtCCAAaTGG	AGCCTGCCTCTCTCTCTAGG	GTCACAAACTGATCCCCGCT
Off target 34	chr12：57594898	+	3	1	tTTCATGGATTtGTtCCCAATGGG	ATGATAGCTTGGCTGGTGGG	TCAAAAATGCAAGCCTCTGGC
Off target 35	chr13：34549231	+	3	1	CTTCTGGCAaTCaTGCaCAATGGG	ATTTCCTTGTCCAGCTGCCAT	CAGCCTCTGGAAGGCTATGT
Off target 36	chr14：105253820	+	3	1	CTTCTTGaATTgtTGCCCAATAGG	GTGCGACACTGTAGTGGTGA	ATCCACTCTGGCAGGAGCTA
Off target 37	chr15：62069433	+	3	1	CTTCTGGcTGTgGTGCCCAgTAGG	CAAGGATGCTGCCTTGGCTA	GTGACGACAGGATTTCGTGC
Off target 38	chr16：82038859	-	3	1	CTTTCTGGATTCcTGtCtAATAGG	TCGTTATTGGCAGCCGATGG	AATTACCCTTCCTGGTTCCAAT TC
Off target 39	chr17：29151412	-	3	1	CTTCTGGtTTgGTGaCCAACTTGG	ATCACAGTGGCCCTAGGAGA	TAAACCAGGCATGGGATGGG
Off target 40	chr18：43341028	-	3	1	CTTTCTGGAcTCtTtCCCAATGGG	CAGAAGGCTAATGGGCGGAA	TGTGCTGTCCACGGTTGTTA

Supplementary Table 4. Online sgRNA Design by Benchling

	Sequence	Specificity Score	Efficiency Score
sgRNA1	ctgggctctaggaattcc	83	64
sgRNA2	cataggctccatagtcct	89	39
sgRNA3	cagaaatcgcaagcatag	78	55
sgRNA4	gcagtctttccaacacaa	71	44
sgRNA5	agtgtgatcacctggtta	91	18
sgRNA6	aaaagttctggaaatgaa	79	56
sgRNA7	tgtatatggtccgtacctg	90	61
