# Supplementary material for: In vivo genome editing via CRISPR/Cas9-mediated homology-independent targeted integration for Bietti crystalline corneoretinal dystrophy treatment
Source: Nat Commun. 2024 May 6;15:3773. doi: 10.1038/s41467-024-48092-9 (PMC11074121; doi:10.1038/s41467-024-48092-9)
Supplement: Supplementary file 3 — Reporting Summary [file 41467_2024_48092_MOESM3_ESM.pdf]

nature portfolio

Corresponding author(s): Liping Yang

Last updated by author(s): Apr 13, 2024

Reporting Summary

Nature Portfolio wishes to improve the reproducibility of the work that we publish. This form provides structure for consistency and transparency in reporting. For further information on Nature Portfolio policies, see our Editorial Policies and the Editorial Policy Checklist.

Statistics

For all statistical analyses, confirm that the following items are present in the figure legend, table legend, main text, or Methods section.

n/a	Confirmed	
☐	☒	The exact sample size (*n*) for each experimental group/condition, given as a discrete number and unit of measurement
☐	☒	A statement on whether measurements were taken from distinct samples or whether the same sample was measured repeatedly
☐	☒	The statistical test(s) used AND whether they are one- or two-sided<br>*Only common tests should be described solely by name; describe more complex techniques in the Methods section.*
☒	☐	A description of all covariates tested
☒	☐	A description of any assumptions or corrections, such as tests of normality and adjustment for multiple comparisons
☐	☒	A full description of the statistical parameters including central tendency (e.g. means) or other basic estimates (e.g. regression coefficient) AND variation (e.g. standard deviation) or associated estimates of uncertainty (e.g. confidence intervals)
☐	☒	For null hypothesis testing, the test statistic (e.g. *F*, *t*, *r*) with confidence intervals, effect sizes, degrees of freedom and *P* value noted<br>*Give P values as exact values whenever suitable.*
☒	☐	For Bayesian analysis, information on the choice of priors and Markov chain Monte Carlo settings
☒	☐	For hierarchical and complex designs, identification of the appropriate level for tests and full reporting of outcomes
☒	☐	Estimates of effect sizes (e.g. Cohen's *d*, Pearson's *r*), indicating how they were calculated

Our web collection on statistics for biologists contains articles on many of the points above.

Software and code

Policy information about availability of computer code

Data collection	The agarose gels were collected by Gel Doc imaging system (Bio- Rad, USA); The PCR amplicons were analyzed by NGS using the Illumina NovaSeq platform (Illumina, San Diego, CA, USA); Fundus photography and optical coherence tomography (OCT) examinations were performed using a Micron IV retinal imaging system (Phoenix-Micron, NW York Drive, USA); ERGs were recorded using the Espion E2 recording system (Diagnosys LLC, Lowell, MA, USA); Immunofluorescence images were captured using a confocal scanning microscope (A+/AIR+, Nikon, Japan). HE staining images were photographed and measured using NanoZoomer Digital Pathology (Hamamatsu Photonics, Hamamatsu City, Japan); RPE flat-mounts images were photographed using a microscope (Nikon, Tokyo, Japan); Western Blot was collected using a ChemiDoc™ MP imaging system (Tanon Science & Technology Co., Shanghai, China); TEM ultrathin sections were viewed on an electron microscope (JEM-1400PLUS, Japan);Real-Time PCR was performed with the ABI7500 Real-Time PCR Detection System (Carlsbad, CA, USA);The OD values were detected with a microplate reader (BioTek Synergy H1, USA); LC-MS/MS targeted peak area was calculated using Target Lynx quantitative software (Waters Corporation);
Data analysis	All data were collected and analyzed using Excel 2019 and performed using Prism Software (GraphPad). The results were analyzed using the student's t test.

For manuscripts utilizing custom algorithms or software that are central to the research but not yet described in published literature, software must be made available to editors and reviewers. We strongly encourage code deposition in a community repository (e.g. GitHub). See the Nature Portfolio guidelines for submitting code & software for further information.

Data

Policy information about availability of data

All manuscripts must include a data availability statement. This statement should provide the following information, where applicable:

- Accession codes, unique identifiers, or web links for publicly available datasets
- A description of any restrictions on data availability
- For clinical datasets or third party data, please ensure that the statement adheres to our policy

The human genome (GRCh38) and mouse genome (GRCm38) data used in this study are available at GenBank Overview (nih.gov). The raw sequencing dataset needed to evaluate the conclusions in this study have been deposited in Figshare. The link is https://doi.org/10.6084/m9.figshare.25568439. All data generated in this study are provided in this manuscript and its Supplementary information/Source Data file.

Research involving human participants, their data, or biological material

Policy information about studies with human participants or human data. See also policy information about sex, gender (identity/presentation), and sexual orientation and race, ethnicity and racism.

Reporting on sex and gender	One BCD patient and one healthy volunteer were enrolled in this study without gender nor sex bias.This study conformed to the tenets of the Declaration of Helsinki.
Reporting on race, ethnicity, or other socially relevant groupings	One BCD patient(homozygous c.802-8_810dell7ins GC) and one healthy volunteer were enrolled in this study, and they were recruited from the Department of Ophthalmology, Peking University Third Hospital. Written informed consent was obtained from all participants, and the ethics committee approved this consent procedure.
Population characteristics	Aside from genetic lineage, the patient also underwent standard clinical ophthalmic examinations, including best-corrected visual acuity, slit-lamp biomicroscopy, dilated indirect ophthalmoscopy, fundus photography, fundus autofluorescence and optical coherence tomography (OCT). The molecular diagnosis of BCD was performed as previously described and the variants were confirmed by Sanger sequencing.
Recruitment	One BCD patient(homozygous c.802-8_810dell7ins GC) and one healthy volunteer recruited from the Department of Ophthalmology, Peking University Third Hospital without bias. The required mutation is inherent and do not change with the population. Additionally, participants received monetary compensation for their participation and unblinding of corresponding experimental data was performed only after completion of collection experiments.
Ethics oversight	Peking University Third Hospital Medical Ethics Committee (No. 2021262)

Note that full information on the approval of the study protocol must also be provided in the manuscript.

Field-specific reporting

Please select the one below that is the best fit for your research. If you are not sure, read the appropriate sections before making your selection.

☒ Life sciences ☐ Behavioural & social sciences ☐ Ecological, evolutionary & environmental sciences

For a reference copy of the document with all sections, see nature.com/documents/nr-reporting-summary-flat.pdf

Life sciences study design

All studies must disclose on these points even when the disclosure is negative.

Sample size	The sample size was determined based on our previous paper that has used similar technology in life science study(elife, 2023). This study was designed to compare the editing therapy across different aspects. The samples were used for DNA/RNA/protein/FFA extraction, histology and cells analysis and morphologic observation. The sample size was selected to ensure minimization of statistical errors. The exact number of analyzed samples is specified for each experiment in the corresponding figure and/or figure legends.
Data exclusions	The mice excluded from the following study are those with unsuccessful subretinal injection. It mainly caused by the surgical process, such as the retinal hemorrhage and vectors delivery failure. In order to exclude errors caused by experimental manipulations, only those animals with successful operation were collected for further study. The number of vitreous hemorrhage mice during the surgery is two. Sixty-four animals with no apparent surgical complications were included for further evaluation. There is no difference between different groups.
Replication	The experiments were performed in two to three independent replicates as properly indicated in the statistics and reproducibility statement. The replications had similar and consistent results in our research.
Randomization	All animals were randomly allocated to experimental groups. The samples used for DNA/RNA/protein/FFA extraction, histology and cells analysis and morphologic observation were randomly chosen.
Blinding	We were blinded to group allocation during data collection and analysis.

Reporting for specific materials, systems and methods

We require information from authors about some types of materials, experimental systems and methods used in many studies. Here, indicate whether each material, system or method listed is relevant to your study. If you are not sure if a list item applies to your research, read the appropriate section before selecting a response.

Materials & experimental systems

n/a	Involved in the study
☐	☒ Antibodies
☐	☒ Eukaryotic cell lines
☒	☐ Palaeontology and archaeology
☐	☒ Animals and other organisms
☒	☐ Clinical data
☒	☐ Dual use research of concern
☒	☐ Plants

Methods

n/a	Involved in the study
☒	☐ ChIP-seq
☒	☐ Flow cytometry
☒	☐ MRI-based neuroimaging

Antibodies

Antibodies used

The primary antibodies used in this study were as follows: rabbit monoclonal anti-NANOG antibody (1:200; ab109250; Abcam, Cambridge, MA), rabbit polyclonal anti-OCT4 antibody (1:200; ab19857; Abcam), mouse monoclonal anti-SSEA4 antibody (1:200; sc-21704; SantaCruz), mouse monoclonal anti-TRA-1-60 antibody (1:400; ab16288; Abcam), rabbit monoclonal anti-CRALBP antibody (1:100; A11649; ABclonal, China), rabbit monoclonal anti-PAX6 antibody (1:100; A7334; ABclonal), rabbit anti-CYP4V2 antibody (1:100; generated by AbMax Biotechnology Co., Ltd.), immunogen CYP4V2 (NP_997235.3,1a.a.-525a.a) full-length human protein; rabbit monoclonal antibody against recombinant human β- actin (1:5000; AC026, ABclonal, Wuhan, China), mouse monoclonal antibody against EGFP (1:1000; ab184601, Abcam, Cambridge, United Kingdom); rabbit Anti-CRISPR-Cas9 antibody (1:5000; ab203943, Abcam, Cambridge, MA); Mouse Monoclonal antibody against α-fetoprotein (AFP) (1:100, MA5-14666,Thermo Fisher); Mouse Monoclonal antibody against α-smooth muscle actin (SMA)(1:200, 14-9760-82,Thermo Fisher); Rabbit Polyclonal antibody against beta Tubulin 3/ TUJ1 (1:200,PA5-85639,Thermo Fisher).

The secondary antibodies were as follows: donkey anti-mouse IgG (H+L), Alexa Fluor 488 (1:800; A21202; Thermo Fisher, Waltham, MA, USA), donkey anti-rabbit IgG (H+L), Alexa Fluor 488 (1:800, A21206, Thermo Fisher), donkey anti-rabbit IgG (H+L), Alexa Fluor 568 (1:800; A10042; Thermo Fisher), donkey anti-mouse IgG (H+L), Alexa Fluor 568 (1:800; A10037, Thermo Fisher); donkey anti-rabbit IgG (H+L), Alexa Fluor 647 (1:800, A31573, Thermo Fisher),; horse radish peroxidase (HRP)-conjugated Goat anti-mouse IgG antibody (1:10005000; , A0216, Beyotime, Shanghai, China) or Goat anti-rabbit IgG antibody (1:5000; A0208, Beyotime).

Validation

The primary antibodies used in this study were validated in the lab, by other groups, and/or details as follows: rabbit monoclonal anti-NANOG antibody (1:200; ab109250; Abcam, Cambridge, MA) [Anti-Nanog antibody (https://www.abcam.com/en-hk/products/primary-antibodies/nanog-antibody-epr20272-ab109250), rabbit polyclonal anti-OCT4 antibody (1:200; ab19857; Abcam) (https://www.abcam.com/en-ph/products/primary-antibodies/oct4-antibody-ab19857), mouse monoclonal anti-SSEA4 antibody (1:200; sc-21704; SantaCruz) (https://www.scbt.com/p/ssea-4-antibody-813-70), mouse monoclonal anti-TRA-1-60 antibody (1:400; ab16288; Abcam) [Anti-TRA-1-60 (R) antibody (https://www.abcam.com/en-ph/products/primary-antibodies/tra-1-60-r-antibody-tra-1-60-ab16288), rabbit monoclonal anti-CRALBP antibody (1:100; A11649; ABclonal, China) (https://abclonal.com/catalog-antibodies/MITFRabbitmAb/A11649), rabbit monoclonal anti-PAX6 antibody (1:100; A7334; ABclonal) (https://abclonal.com/catalog-antibodies/PAX6RabbitpAb/A7334), rabbit anti-CYP4V2 antibody (1:100; generated by AbMax Biotechnology Co., Ltd.), immunogen CYP4V2 (NP_997235.3,1a.a.-525a.a). The antibody was verified in WB, ICC, IHC-P and IHC-Fr, which were provided in the peer review file. (https://www.devex.com/organizations/abmax-biotechnology-147235); rabbit monoclonal antibody against recombinant human β- actin (1:5000; AC026, ABclonal, Wuhan, China) (https://abclonal.com/catalog-antibodies/ActinRabbitmAbHighDilution/AC026), mouse monoclonal antibody against EGFP (1:1000; ab184601, Abcam, Cambridge, United Kingdom) [Anti-EGFP antibody(https://www.abcam.com/en-ph/products/primary-antibodies/egfp-antibody-f56-6a123-ab184601); rabbit Anti-CRISPR-Cas9 antibody (1:5000; ab203943, Abcam, Cambridge, MA) [Anti-CRISPR-Cas9 antibody(https://www.abcam.com/en-ph/products/primary-antibodies/crispr-cas9-antibody-epr19795-ab203943), Mouse Monoclonal antibody against α-fetoprotein (AFP) (1:100, MA5-14666,Thermo Fisher);(https://www.thermofisher.cn/cn/zh/antibody/product/AFP-Antibody-clone-P5B8-Monoclonal/MA5-14666), Mouse Monoclonal antibody against α-smooth muscle actin (SMA)(1:200, 14-9760-82,Thermo Fisher)(https://www.thermofisher.cn/cn/zh/antibody/product/AFP-Antibody-clone-P5B8-Monoclonal/MA5-14666), Rabbit Polyclonal antibody against beta Tubulin 3/ TUJ1 (1:200,PA5-85639,Thermo Fisher)(https://www.thermofisher.cn/cn/zh/antibody/product/beta-3-Tubulin-Antibody-Polyclonal/PA5-85639)

The secondary antibodies were as follows: donkey anti-mouse IgG (H+L), Alexa Fluor 488 (1:800; A21202; Thermo Fisher, Waltham, MA, USA) (https://www.thermofisher.cn/cn/zh/antibody/product/Donkey-anti-Mouse-IgG-H-L-Highly-Cross-Adsorbed-Secondary-Antibody-Polyclonal/A-21202), donkey anti-rabbit IgG (H+L), Alexa Fluor 488 (1:800, A21206, Thermo Fisher)(https://www.thermofisher.cn/cn/zh/antibody/product/Donkey-anti-Rabbit-IgG-H-L-Highly-Cross-Adsorbed-Secondary-Antibody-Polyclonal/A-21206), donkey anti-rabbit IgG (H+L), Alexa Fluor 568 (1:800; A10042; Thermo Fisher) (https://www.thermofisher.cn/cn/zh/antibody/product/Donkey-anti-Rabbit-IgG-H-L-Highly-Cross-Adsorbed-Secondary-Antibody-Polyclonal/A10042), donkey anti-mouse IgG (H+L), Alexa Fluor 568 (1:800; A10037, Thermo Fisher)(https://www.thermofisher.cn/cn/zh/antibody/product/Donkey-anti-Mouse-IgG-H-L-Highly-Cross-Adsorbed-Secondary-Antibody-Polyclonal/A10037), donkey anti-rabbit IgG (H+L), Alexa Fluor 647 (1:800; A31573; Thermo Fisher) (https://www.thermofisher.cn/cn/zh/antibody/product/Donkey-anti-Rabbit-IgG-H-L-Highly-Cross-Adsorbed-Secondary-Antibody-Polyclonal/A-31573), horse radish peroxidase (HRP)-conjugated Goat anti-mouse IgG antibody (1:5000; A0216, Beyotime, Shanghai, China)(https://www.beyotime.com/product/A0216.htm)or Goat anti-rabbit IgG antibody (1:5000; A0208, Beyotime) (https://www.beyotime.com/product/A0208.htm).

Eukaryotic cell lines

Policy information about cell lines and Sex and Gender in Research

Cell line source(s)	HEK293Tcells(CRL-3216) and NIH/3T3 cells(CRL-1658)were purchased from ATCC. Two iPS cell lines(WT and CYP4V2 mutant) was generated in house (laboratory of Beijing Chinagene Co., LTD, Beijing, China) as described in the Methods section and in the publication cited in that section. One healthy male and one female BCD urines (ReprocellUSA) were reprogrammed using the reproEasy iPSC Reprogramming Kit (CA5002002, Cellapy Biotechnology, Beijing, China)according to the manufacturer's protocol.
Authentication	HEK293Tcells(CRL-3216) and NIH/3T3 cells(CRL-1658) purchased from ATCC and was confirmed by morphology.All iPSC lines were karyotyped, genotyped using Sanger sequencing, and tested for pluripotency markers using immunofluorescence staining.
Mycoplasma contamination	All cell lines used in this study tested negative for mycoplasma contamination.
Commonly misidentified lines (See ICLAC register)	No misidentified line included.

Animals and other research organisms

Policy information about studies involving animals; ARRIVE guidelines recommended for reporting animal research, and Sex and Gender in Research

Laboratory animals	All mice were bred and maintained at the Peking University Health Science Center Animal Care Services Facility in Specific Pathogen Free (SPF) conditions under a 12-hour light/12-hour dark cycle with ad libitum access to food and water.The h-Cyp4v3mut/mut mice used in this study were custom designed and obtained from Beijing Biocytogen Co., Ltd (Beijing, China). These models were generated by CRISPR/Cas9 technology based on homology directed repair (HDR). In this mouse model, the mon 6 (mE6) to exon 8 (mE8) was replaced with the human counterpart that contained two common clinical mutations: E7: c.802-8_810del17insGC and E8: c.992A>C simultaneously. A pair of sgRNAs targeting exon 6-8 of the Cyp4v3 gene and a homologous recombination template were designed. The sequence of sgRNAs are as follows, sgRNA for exon 6: 5′-AGAAGGACGGGACCACAAAA-3′; sgRNA for exon 8: 5′-CTTCTGGATTCGTGCCCAAT-3′. The mRNA of in vitro transcribed Cas9 and sgRNAs along with homologous recombination template were injected into zygotes of C57BL/6J mice. After injection, the zygotes were implanted into the oviduct of pseudopregnant mice, and founder mice were obtained by natural birth. The obtained founder mice were validated by PCR, RT-PCR, Western blot for the targeted genome, transcription and translation. The model was also excluded from the retinal degeneration mutations (Pde6brd1, Crb1rd8, Pde6brd10, and Rpe65rd12) using sanger sequencing and potential off-target sites through Cas-OFFinder.
Wild animals	The study did not involve wild animals
Reporting on sex	Mice were randomly distributed to experimental groups, with half males and half females.
Field-collected samples	All mice were bred and maintained at the Peking University Health Science Center Animal Care Services Facility in Specific Pathogen Free (SPF) conditions under a 12-hour light/12-hour dark cycle. Food and water were available ad libitum. All animals were maintained in accordance with the guidelines of the Association for the Assessment and Accreditation of Laboratory Animal Care. All experiments were performed in accordance with the Association for Research in Vision & Ophthalmology (ARVO) Statement for the Use of Animals in Ophthalmic and Vision Research. Mice were randomly distributed to experimental groups, with half males and half females.
Ethics oversight	Peking University Health Science Center Ethics Committee for Experimental Animal Research

Note that full information on the approval of the study protocol must also be provided in the manuscript.

Plants

Seed stocks	*Report on the source of all seed stocks or other plant material used. If applicable, state the seed stock centre and catalogue number. If plant specimens were collected from the field, describe the collection location, date and sampling procedures.*
Novel plant genotypes	*Describe the methods by which all novel plant genotypes were produced. This includes those generated by transgenic approaches, gene editing, chemical/radiation-based mutagenesis and hybridization. For transgenic lines, describe the transformation method, the number of independent lines analyzed and the generation upon which experiments were performed. For gene-edited lines, describe the editor used, the endogenous sequence targeted for editing, the targeting guide RNA sequence (if applicable) and how the editor was applied.*
Authentication	*Describe any authentication procedures for each seed stock used or novel genotype generated. Describe any experiments used to assess the effect of a mutation and, where applicable, how potential secondary effects (e.g. second site T-DNA insertions, mosiacism, off-target gene editing) were examined.*
